# Draft Genome Sequence of *Mycobacterium vulneris* DSM 45247^T^

**DOI:** 10.1128/genomeA.00370-14

**Published:** 2014-05-08

**Authors:** Olivier Croce, Catherine Robert, Didier Raoult, Michel Drancourt

**Affiliations:** Aix Marseille Université, URMITE, Marseille, France

## Abstract

We report the draft genome sequence of *Mycobacterium vulneris* DSM 45247^T^ strain, an emerging, opportunistic pathogen of the *Mycobacterium avium* complex. The genome described here is composed of 6,981,439 bp (with a G+C content of 67.14%) and has 6,653 protein-coding genes and 84 predicted RNA genes.

## GENOME ANNOUNCEMENT

*Mycobacterium vulneris* is a nontuberculous mycobacterium recently individualized among the *Mycobacterium avium* complex (1). The name was given after the initial isolation of the organism from a dog-bite wound discharge; a second isolate was made from a diseased lymph node in a 2-year-old child (1, 2). However, no further isolates have been obtained, so the clinical spectrum of this pathogen as well as its reservoir and sources are not yet known. *M. vulneris* is a mycobacterium previously referred to as *M. avium* sequevar Q (1, 2). *M. vulneris* was shown to be more closely related to *Mycobacterium colombiense*, another member of the *M. avium* complex (1, 3). Its precise relationships with several species recently described in the *M. avium* complex remain unknown (4).

In order to further decrypt the phylogenetic relationships of *M. vulneris* within the *M. avium* complex, we sequenced the whole genome of the *M. vulneris* DSM 45247^T^ strain.

Genomic DNA was isolated from an *M. vulneris* DSM 45247^T^ strain grown on MGIT Middlebrook broth (Becton Dickinson, Sparks, MD) at 37° C. Genomic DNA of *M. vulneris* was sequenced on MiSeq Technology (Illumina, Inc., San Diego, CA) by using paired-end and mate-pair applications in parallel, in a 2- × 250-bp run for each bar-coded library. On each flow cell, The index representation for *M. vulneris* was determined to be 5.46% and 7.71%, respectively, on each flow cell. The total 1,697,812 reads were filtered according to the read qualities.

The whole set of reads was trimmed using Trimmomatic (5), and then assembled with the assembler software Spades v 3.0 (6, 7). Contigs obtained were combined together by SSPACE v 2.0 (8) and Opera software v 1.4 (9) and helped by GapFiller v 1.10 (10) to reduce the set. For some manual refinements we used the CLC Genomics v 6 software (CLC bio, Aarhus, Denmark) and homemade tools. The completed draft genome sequence of *M. vulneris* consists of four contigs without gaps, containing 6,981,439 bp and 67.14% G+C content.

Noncoding genes and miscellaneous features were predicted using RNAmmer (11), ARAGORN (12), Rfam (13), and PFAM (14). Open reading frames (ORFs) were predicted using Prodigal (15), and functional annotation was achieved using BLASTP against the GenBank database (16) and the Clusters of Orthologous Groups (COGs) database (17, 18). The genome was shown to encode at least 84 predicted RNAs, including 7 rRNAs, 58 tRNAs, 1 transfer-messenger RNA (tmRNA), and 18 miscellaneous RNAs. A total of 6,653 genes were also identified, representing a coding capacity of 6,470,571 bp and a 92.6% coding percentage. Whereas 6,608 genes matched a least one sequence in the COGs database when BLASTP default parameters were used, 881 (13.24%) genes encoded putative proteins and 1,051 (15.8%) genes were assigned as hypothetical proteins.

### Nucleotide sequence accession numbers.

The *Mycobacterium vulneris* strain DSM 45247^T^ genome sequence has been deposited at EMBL under the accession numbers CCBG010000001 through CCBG010000004.
